# Monitoring lung tumour volume on daily cone beam CT; is it achievable in a real-world setting?

**DOI:** 10.1016/j.tipsro.2025.100352

**Published:** 2025-10-23

**Authors:** Sarah Barrett, Laure Marignol, Gerard G Hanna, Conor K McGarry, Gerard M Walls

**Affiliations:** a Applied Radiation Therapy Trinity, Trinity College Dublin, Dublin, Ireland; b Trinity St. James’s Cancer Institute, Trinity College Dublin, Dublin, Ireland; c St. Luke’s Radiation Oncology Network, St. Luke’s Hospital Rathgar, Dublin, Ireland; d Patrick G. Johnston Centre for Cancer Research, Queen’s University Belfast, Belfast, United Kingdom; e Northern Ireland Cancer Centre, Belfast Health & Social Care Trust, Belfast, United Kingdom

## Abstract

Delineating the gross tumour volume (GTV) in non-small cell lung cancer (NSCLC) can be challenging due to anatomical complexities and imaging artefacts. This study evaluates a semi-automated workflow using commercial software for target volume (TV) delineation on cone-beam CT (CBCT) in patients undergoing radical radiotherapy. Seventy-six patients with 553 scans (n = 76 planning CT, n = 477 CBCT) were included. Auto-contours were adjusted by a senior Radiation Therapist and reviewed for accuracy. The majority (59.1 %) required only minor revisions, with median adjustment time of CBCT auto-contours of 83 (range 0–460) seconds. The findings support the feasibility of this approach, offering a pragmatic solution for adaptive radiotherapy workflows in NSCLC.

## Highlights

• 66 % of patient scans were suitable for lung tumour delineation on 3DCBCT.
• Adjustment of auto-contours on CBCT was efficient taking median of 83 (range 0–460) seconds.
• Internal audit of generated volumes showed excellent agreement between RO and RTT.

## Introduction

Delineation of the gross tumour volume (GTV) is challenging in non-small cell lung cancer (NSCLC) owing to cardiopulmonary motion artefact, proximity of mediastinal structures with similar density, and acute changes such as atelectasis and infective consolidation [1]. The accuracy of segmentation of the GTV during radiation therapy (RT) planning can be improved with the utilization of 4 Dimensional Computed Tomography (4DCT), Intravenous (IV) contrast and ^18^Fluorodeoxyglucose Positron Emission Tomography (FDG-PET) CT and is recommended in international lung cancer radiotherapy contouring guidelines [1,2].

With the advent of adaptive RT (ART), repeated definition of the target volume (TV) on cone-beam CT (CBCT) is necessary and developing guidelines in this area is paramount as online ART increasingly becomes a realizable clinical workflow [3]. Performing these delineations manually is resource intensive and inefficient as this requires the expertise and time of a Radiation Oncologist (RO) or specialist Radiation Therapist (RTT). Alternatively, software can be used to deform the original GTV onto the CBCT with the clinician editing the deformed contour [4,5]. These solutions are both resource-intense and unrealistic for large-scale repeated delineation, and a more widely accessible, pragmatic alternative is desirable.

Herein we describe our experience of semi-automated TV delineation in NSCLC on CBCTs using a commercially available solution (Smart Segmentation, Varian Medical Systems, Inc., Palo Alto, CA), with the aim of identifying factors that may influence the feasibility of this workflow in a cohort of patients treated with radical RT for NSCLC.

## Materials and methods

### Patient population

Governance approvals were provided and ethical approval waived, by the Belfast Health & Social Care Trust (IRAS 293181) for this study. The NI-HEART database of 478 patients with NSCLC treated with radical RT was accessed to select patients for CBCT delineation [6]. Initial screening to identify patients with T1, 2, 3 primary tumours and N0 M0 disease excluded 352 cases. Node-positive patients were excluded as this study sought to examine primary tumour GTV delineation only. A further 12 were excluded due to miscoding of T or N stage, or missing DICOM data. All patients were treated with 55 Gy/20# RT alone.

**Fig. 1:**
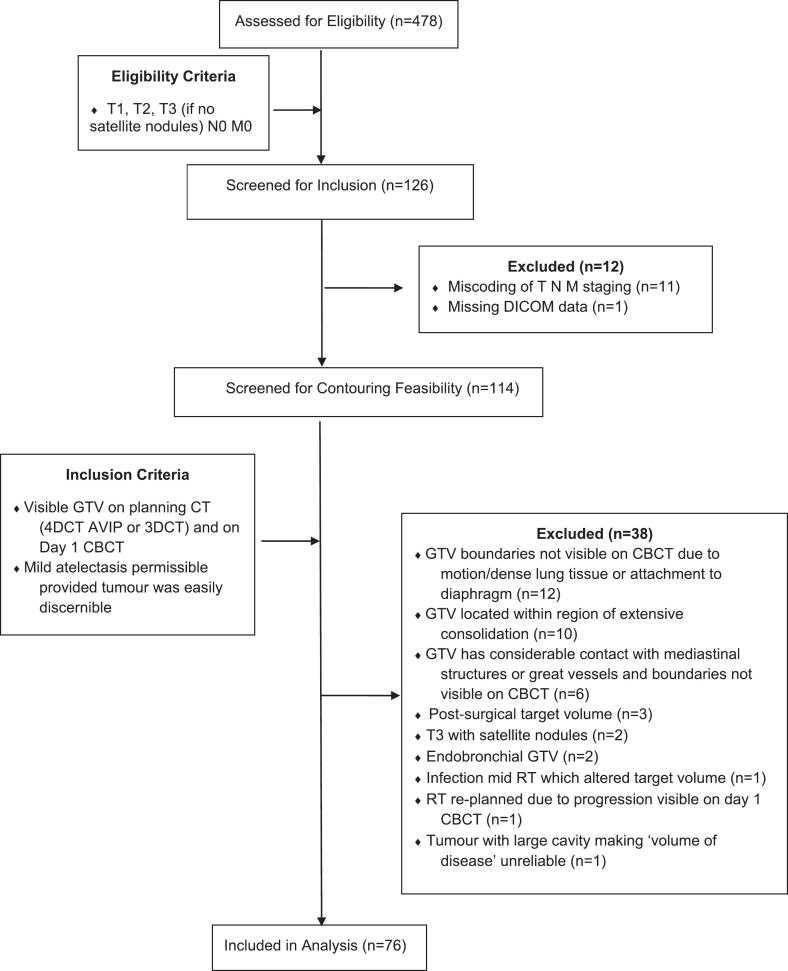
Participant flow diagram outlining inclusion and exclusion criteria, GTV: Gross Tumour Volume, 4DCT: 4-Dimensional Computed Tomography, CBCT: Cone Beam Computed Tomography, AVIP: Average Intensity Projection, 3DCT 3- Dimensional Computed Tomography, RT: Radiation Therapy, DICOM: Digital Imaging and Communications in Medicine.

A total of 114 patient scans were anonymised and the planning CT average image projection (AVIP) reconstruction of the 4DCT, and day 1 CBCT, were screened for suitability to define the TV. Patients were excluded as per criteria developed in Fig. 1. For each eligible patient, a pre-treatment planning 3DCT or 4DCT were included, as well as CBCT days 1–3 and a weekly CBCT thereafter (minimum 3 further CBCTs). Planning CT scans were acquired on a GE large bore Optima 580, Discovery CT590 RT (GE Healthcare, Waukesha, WI) or a Siemens SOMATOM. CBCT scans were acquired on Varian TrueBeam™ (Varian Medical Systems, Palo Alto, California, USA) imaging panels and the default Varian Lung protocol without adjustment to the manufacturer kV or mA.

### GTV segmentation

For each patient the planning AVIP or 3DCT was assessed and the internal target volume defined for treatment planning was reviewed in both lung and abdominal window settings. Each tumour was assessed for abutment of normal structures (chest wall, diaphragm, mediastinum (above heart chambers), mediastinum (below heart chambers), apex of lung or other). The percentage of TV effacing normal soft tissue was recorded using a 4-point qualitative scale (Minimal contact, <25 %, 25–50 %, 50–75 %).

On the planning scan, an auto-contour (AC) was generated using the lung tumour Smart Segmentation function on Varian Eclipse™ V18.0 following the manufacturer’s instructions (Supplementary Materials, Fig. 1). Lung windows were utilised to manually identify the required approximate craniocaudal mid-volume CT slice.

The AC was duplicated and manually adjusted by a senior RTT (SB) to exclude gross extensions into adjacent normal tissues to generate a user-adjusted auto-contour (UA-AC). Windowing presets of lung (0 HU to −1000 HU) and abdominal (225 HU to −125 HU) were used during manual adjustment for visualisation of tumour boundaries in contact with lung tissue, or soft tissue structures respectively. This process was repeated for each subsequent CBCT resulting in an AC and UA-AC of the TV for each scan. Time required to adjust the AC volumes was recorded in seconds. For each patient with a 4DCT planning scan, a value representative of tumour motion magnitude was derived as a measure of distance between the centre of mass of the clinical GTV on an expiration 4DCT phase (50 %) and clinical GTV on inspiration phase (0 %). Actual and absolute volume differences between the AC and the UA-AC were measured to quantify the extent of revision required.

### Contour evaluation

A selection of the UA-AC volumes were audited by a senior Clinical Oncology Research Fellow (GW) in the Eclipse contouring workspace. A random sampling approach was used to select contours for blinded review to evaluate UA-AC on planning CT and the available CBCTs. Contours were rated with a 4-point scale (1 = no revisions, 2 = minor revisions, 3 = major revision, 4 = unsuitable contour) in line with the most commonly reported qualitative evaluation scales [7].

### Statistical analysis

Descriptive analysis of the contour analysis metrics including frequency of ratings, percentages of tumours effacing soft tissue, and median values of adjustment time were reported. To ascertain if a time trend existed in contouring the first to the last CBCT, a single-factor Analysis of Variance (ANOVA) was conducted. Data were tested for normality and a Wilcoxon Signed Rank test was used to assess the significance of any difference in the AC adjustment times between planning CT and both day 1 and final CBCTs. P values < 0.05 were interpreted as equating with statistical significance. Per patient average time to adjust contours will be plotted against tumour motion and percentage of tumour adjacent to soft tissue to assess for correlations.

## Results

### Patient population

Following screening of n = 114 cases, 33 % (n = 38) were excluded, resulting in 76 included patients. The most common rationale for exclusion was the TV boundary not being easily discernible on CBCT due to motion-related artefact and/or tumour location. See Fig. 1 for exclusion reasons and Supplementary Materials for representative images of excluded cases.

As a result, n = 553 scans were contoured: n = 72 AVIP, n = 4 3D planning CT and n = 477 CBCT. See Supplementary Materials, Table 2, for demographic details on included patients. Patients had an average of 7 scans in total (range 6–10).

### Target volume segmentation and evaluation

**Fig. 2:**
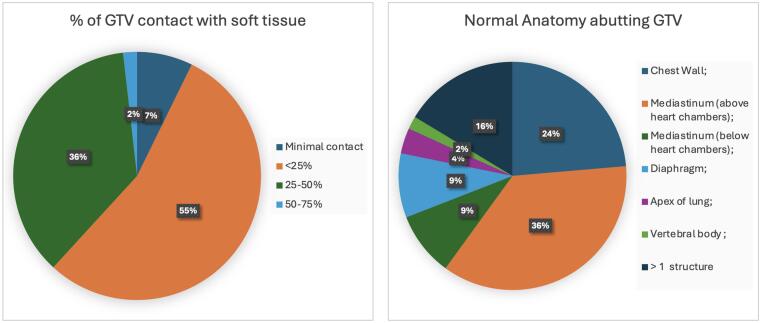
Summary of tumour contact with normal soft tissue structures (n = 55 patients) (GTV: Gross Tumour Volume) Panel (a) showing the percentage of the GTV in contact with soft tissue Panel (b) notes the breakdown of various anatomical stru*ctures in contact with the GTV.*

The majority (71 %, n = 55) of tumours adjoined ≥1 soft tissue structure, and the distribution of adjacent structures are shown in Fig. 2 Of the 72 patients who had a 4DCT, median tumour motion was 0.3 cm (range 0–2.4 cm).

**Fig. 3:**
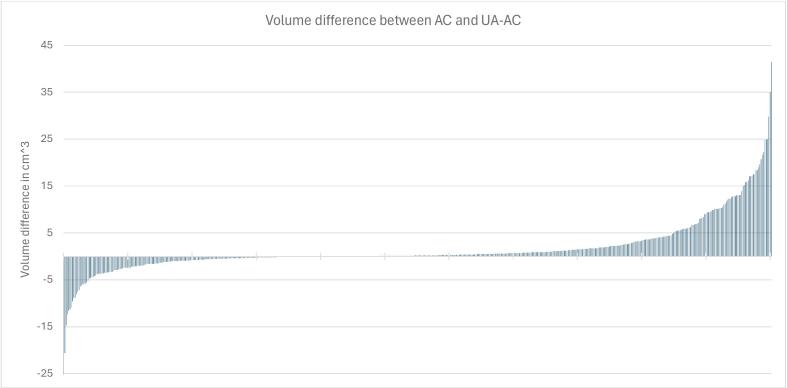
Volume difference between auto-contour and adjusted auto-contour for each scan, arranged by magnitude difference (excluding one outlier where the difference was −100 cm^3^).Median difference 0.2 cm^3^ range −100–41.50.2 cm^3^.

No revision of the AC was required in 8.1 % (n = 45) of the scans, minor revisions were necessary in 59.1 % (n = 327), major revisions in 30.7 % (n = 170) and 2 % (n = 11) of AC volumes were deemed unsuitable for editing. Median volume difference between AC and UA-AC was 0.9 cm^3^ (range 0–100 cm^3^), (Fig. 3).

Across all scans, the median time to adjust the AC on the planning CT was 59 s (range 0–501 s) and on CBCT was 83 s (0–460 s). Day 1 CBCT was significantly longer than planning CT, median of 92 s (range 84–460 s), *p* < 0.0001. There was no significant difference in contour adjustment time from first CBCT to last, median 98 s (range 92–298 s), *p* = 0.79. Median adjustment time for adenocarcinoma and squamous carcinoma subtypes were 86.5 s (range 6.6–166.4 s) and 112.7 (33.9–292.1 s) respectively, (*p* = 0.123).

**Fig. 4:**
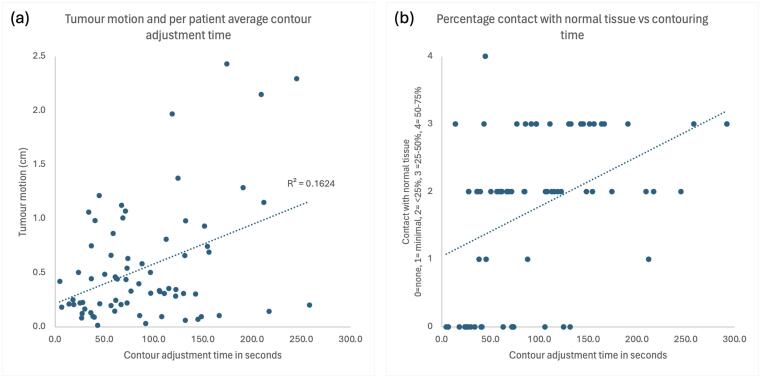
Scatter plots demonstrating the relationship between contour adjustment time and tumour motion and tumour c*ontact with normal tissue.*

For each patient an average time to adjust the AC was calculated and this was plotted against representative tumour motion, and the percentage of the GTV touching soft tissue. Fig. 4 shows a positive correlation between an increased contour adjustment time with each factor were observed, although not statistically significant (Fig. 4).

17 % (n = 102) of scans were included in a blinded, independent audit, and excellent agreement was observed between the RO and RTT UA-ACs (Supplementary Materials, Table 3) Only two volumes required major revision and these revisions occurred in the same patient where a small extension of tumour was omitted by the auto-contouring system and was also not included in the adjusted segmentation.

## Discussion and Conclusion

This study sought to identify the feasibility of semi-automated TV delineation of NSCLC cases on CBCT and to elucidate relevant factors which may influence this process. A significant percentage of patients (39.6 %) were unsuitable for CBCT delineation primarily due to challenges defining the TV on non-contrast enhanced imaging. The benefit of both IV contrast [8] and nuclear imaging such as FDG-PET [2] are well established recommendations to improve TV delineation in planning CT scans. The lack of these imaging aids in CBCT comparatively restricts tumour visualisation on these scans. More recently, improvements in CBCT technology through faster acquisition times, larger fields of view, enhanced contrast resolution and artifact suppression have improved CBCT imaging quality, which will improve the delineation on CBCT [9].

Except for tumours in broad contact with the diaphragm or the hila, effacement of normal soft tissue structures did not prevent TV delineation on CBCT. While a trend for increased contouring time was observed in those cases, the increase was not significant, however a limitation here was the subjective assessment of tumour effacement. Similarly, the increased contour adjustment time noted for tumours with greater motion was not significant. In all cases, the overall time required to manually adjust the AC was low, median 83 s on CBCT, suggesting that this workflow may be suitable for adoption for select cases on busy treatment units. While time saving is reported in AC studies, there is a paucity of data on manual adjustment time required to edit AC volumes [4,10]. Furthermore timesaving needs to be considered in the context of optimised workflows to ensure the benefits translate to increased efficiencies for patients [11].

The delineation of TVs in patients with NSCLC is subject to uncertainties due to the impact of cardiopulmonary motion and image resolution limitations [12,13] and, as no ‘ground truth’ GTV delineation on CBCT is available for formal evaluation of contour accuracy, the clinician’s manual contour remains the gold standard. Comparison to the physician-defined planning GTV was not possible as this defined on a different dataset, usually including all 10 phases of the 4DCT and/or the maximum intensity projection reconstruction. In this study excellent agreement between the RO and RTT was seen, with only 2 scans requiring a major revision. Prior work using this contouring approach on 4DCT data demonstrated both geometric and dosimetric acceptability of the GTV volumes generated [14]. The main limitation of this work is that a single user performed all volume adjustments, meaning inter-observer variability was not evaluable. In addition a single expert audited the resulting contours. Follow-up confirmatory work currently in planning will incorporate multiple observers of various seniority levels. AI informed tools to generate TVs are available, but the majority of commercial solutions focus on OAR delineation with a recent review highlighting only 13 of 40 included studies reported on TV [10]. One study reporting on automated and semi-automated GTV delineation for adaptive RT in the locally advanced NSCLC setting reported the automated contours to be generally acceptable, with minimal changes required to the AC however larger modifications were required when there was greater tumour regression observed [15].

A 2020 survey highlighted that increasing ART in lung cancer RT is a priority for many centres, but technical limitations were identified as a barrier [5]. One such technical limitation is CBCT image quality restricting TV definition. The auto-segmentation workflow described in this study was robust to this challenge however, and therefore could be implemented in centres establishing online ART pathways. In this scenario, clinical accuracy is critical and more extensive validation would be needed to verify the validity of this approach. Under these circumstances, the RTT adjusting the contours would work within an Advanced Practice framework, and thorough assessment and credentialling would be essential to ensure the veracity of the contours generated. Advanced Practice RT roles have demonstrated that this is feasible, and is associated with enhanced efficiency in the clinical workflow [16].

Alternatively, the tool could be used as an efficient on-treatment check of TV regression or progression, to quantify substantial changes and flag the need for a re-scan and re-plan [17]. In this situation, we posit that RTTs, without advanced practice competences could deploy the tool with sufficient training and supervision task. The workflow presented here suggests manual edits off normal anatomy are required, not the generation of a target volume without any guidance. Furthermore, integration of such a system in routine care would enable the generation of an online TV for future large real-world research datasets [18].

In summary, this study has identified factors associated with suitability for CBCT TV delineation in a real-world setting. In eligible patients, we have shown this workflow, using a commercially available solution, produced good quality TVs in a short timeframe.

## Declaration of competing interest

The authors declare the following financial interests/personal relationships which may be considered as potential competing interests: A research grant from Varian Medical Systems, (grant number 011871), partially funded this work to S.B. and L.M. Varian Medical Systems had no input in this study design, data analysis, or manuscript preparation.
