# Supplementary material for: Monitoring lung tumour volume on daily cone beam CT; is it achievable in a real-world setting?
Source: Tech Innov Patient Support Radiat Oncol. 2025 Oct 23;36:100352. doi: 10.1016/j.tipsro.2025.100352 (PMC12616081; doi:10.1016/j.tipsro.2025.100352)
Supplement: Supplementary Data 1 [file mmc1.docx]

Supplementary Materials

Figure 1 Manufacturer’s instructions on Lung Tumour Segmentation


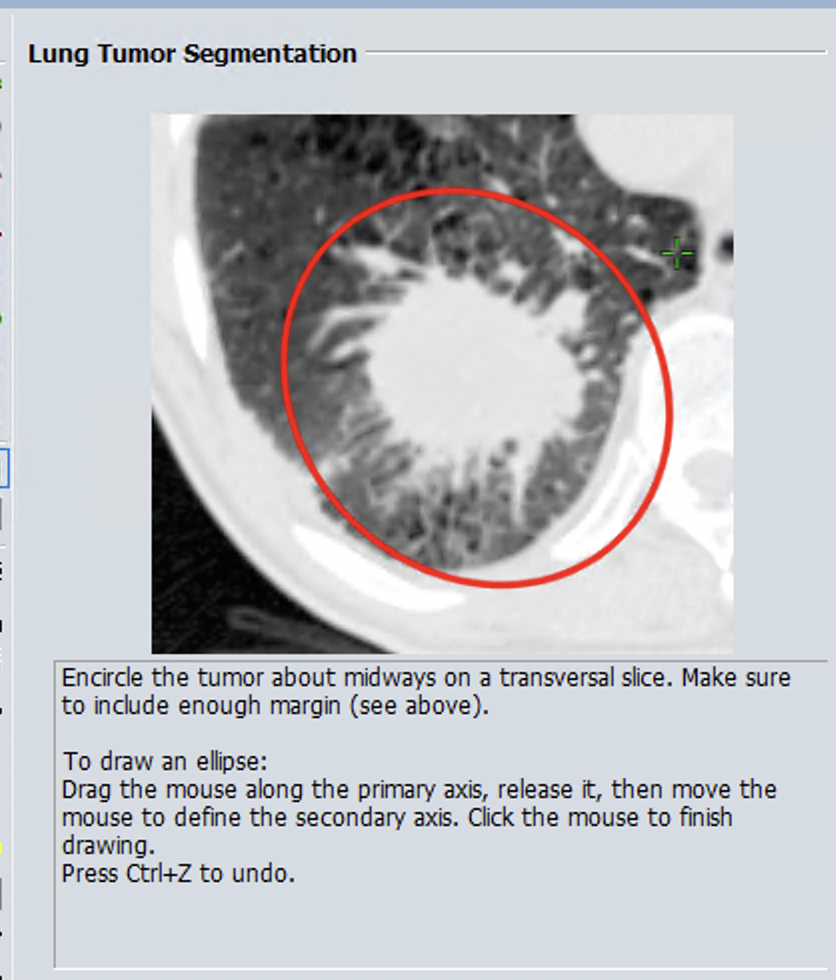


Figure 2 Sample images of excluded and included patients

Tumour in right lower lobe with large motion making sup border indistinct and attached to diaphragm resulting in unclear inferior border.

Tumour in right lower lobe attached with large motion and dense lung tissue making all GTV boundaries indistinct.

Tumour in left hilum where IV contrast aided delineation at planning CT (right) but borders were not clear on CBCT (left).

Example where auto-contouring failed to identify the tumour due to artefact on the scan due to large body habitus.

**

Example where auto-contouring performed well and minimal adjustments were required

Table 1 Demographics

| Patient Demographics | N (%) |
| --- | --- |
| Age (median, range) years | 73.5 (50-90) |
| Gender |  |
| Female | 45 (59%) |
| Male | 31 (41%) |
| Performance Status |  |
| 0 | 5 (6.6%) |
| 1 | 27 (35.5%) |
| 2 | 39 (51.3%) |
| 3 | 5 (6.6%) |
| T-Stage |  |
| 1 | 38 (50%) |
| 2 | 21 (27.6%) |
| 3 | 17 (22.4%) |
| Histological Subtype |  |
| Squamous cell carcinoma | 25 (32.9%) |
| Adenocarcinoma | 14 (18.4%) |
| Clinical | 34 (44.7%) |
| Other | 3 (4%) |
| Dose Fractionation Schedule |  |
| 52–55Gy/19–20# | 72 (94.7%) |
| 60–66Gy/30–33# | 4 (5.3%) |
| Radiotherapy planning |  |
| 3DCRT | 24 (31.6%) |
| IMRT | 14 (18.4%) |
| VMAT | 38 (50%) |

*3DCRT: 3 Dimensional Conformal Radiation Therapy, IMRT: Intensity Modulated Radiation Therapy, VMAT: Volumetric Modulated Arc Therapy*

Table 2 Results of the independent audit of user-adjusted auto contours

|  | (n) | % |
| --- | --- | --- |
| Total reviewed | 102 | 100 |
| No revisions | 87 | 85.3 |
| Minor revisions | 13 | 12.7 |
| Major revision | 2 | 2.0 |
| Unsuitable contour | 0 | 0.0 |
